# Comb-Push Ultrasound Shear Elastography of Breast Masses: Initial Results Show Promise

**DOI:** 10.1371/journal.pone.0119398

**Published:** 2015-03-16

**Authors:** Max Denis, Mohammad Mehrmohammadi, Pengfei Song, Duane D. Meixner, Robert T. Fazzio, Sandhya Pruthi, Dana H. Whaley, Shigao Chen, Mostafa Fatemi, Azra Alizad

**Affiliations:** 1 Department of Physiology and Biomedical Engineering, Mayo Clinic College of Medicine, Rochester, Minnesota, United States of America; 2 Department of Radiology, Mayo Clinic College of Medicine, Rochester, Minnesota, United States of America; 3 Department of Internal Medicine, Mayo Clinic College of Medicine, Rochester, Minnesota, United States of America; The University of Queensland, AUSTRALIA

## Abstract

**Purpose or Objective:**

To evaluate the performance of Comb-push Ultrasound Shear Elastography (CUSE) for classification of breast masses.

**Materials and Methods:**

CUSE is an ultrasound-based quantitative two-dimensional shear wave elasticity imaging technique, which utilizes multiple laterally distributed acoustic radiation force (ARF) beams to simultaneously excite the tissue and induce shear waves. Female patients who were categorized as having suspicious breast masses underwent CUSE evaluations prior to biopsy. An elasticity estimate within the breast mass was obtained from the CUSE shear wave speed map. Elasticity estimates of various types of benign and malignant masses were compared with biopsy results.

**Results:**

Fifty-four female patients with suspicious breast masses from our ongoing study are presented. Our cohort included 31 malignant and 23 benign breast masses. Our results indicate that the mean shear wave speed was significantly higher in malignant masses (6 ± 1.58 m/s) in comparison to benign masses (3.65 ± 1.36 m/s). Therefore, the stiffness of the mass quantified by the Young’s modulus is significantly higher in malignant masses. According to the receiver operating characteristic curve (ROC), the optimal cut-off value of 83 kPa yields 87.10% sensitivity, 82.61% specificity, and 0.88 for the area under the curve (AUC).

**Conclusion:**

CUSE has the potential for clinical utility as a quantitative diagnostic imaging tool adjunct to B-mode ultrasound for differentiation of malignant and benign breast masses.

## Introduction

Conventional B-mode ultrasound (US) is commonly used to differentiate benign and malignant breast masses [1,2,3,4]. Although B-mode US is a valuable adjunct to mammography, which improves diagnostic sensitivity, it does suffer from low specificity [5,6,7,8,9] leading to a large number of unnecessary benign biopsies [10]. Currently, the high rate of benign biopsies performed in the United States results in significant financial and emotional burden for the patient, and a significant allocation of healthcare dollars. An additional ultrasound tool to improve specificity in the characterization and classification of breast masses would help reduce the number of unnecessary benign biopsies.

Elasticity medical imaging is an emerging field that provides palpation-like information such as a tissue’s stiffness [11]. It is well known that malignant breast masses are usually stiffer than benign masses [12,13]. Therefore, techniques that can noninvasively assess a tissue’s pathology based on its mechanical properties can improve disease diagnosis [14,15]. One such technique, quasi-static elastography, which is based on the relative deformation of the tissue or strain, has recently been reported to increase the specificity of B-mode US in differentiating between benign and malignant breast masses [14]. However, quasi-static elastography techniques are not quantitative tools and their user dependency may hamper their clinical value [16]. An alternative is the newly emerging shear wave elastography techniques, which use acoustic radiation force to generate shear waves and quantify tissue elasticity from measured shear wave speed. Because shear waves travel more slowly in softer tissue and faster in stiffer tissues, shear wave elastography could be used for characterization and classification of breast masses [17].

The Shear Wave Elasticity Imaging (SWEI) is first introduced by Sarvazyan et al. [18]. The use of transient shear waves for tissue characterization were first performed by using the transient elastography technique in 1D in 2D and also by using acoustic radiation force. The transient elastography technique was tested in breast to detect and classify breast lesions [19,20].

The two most well-known shear wave elastography techniques are shear wave imaging using Acoustic Radiation Force Impulse (ARFI) [21], and Supersonic Shear Imaging (SSI) [17,22]. The ARFI shear wave method employs an acoustic impulse focused within the tissue where the deformation of the tissue generates shear waves that are detected. The measured shear wave speeds are used to calculate the stiffness properties of the tissue [21]. The diagnostic value of shear wave imaging using ARFI for characterization of B-mode detected breast lesions has been studied [23,24,25]. The push method used in SSI relies on generating a supersonic regime moving-source generating shear waves in the tissue using a conventional transducer. To capture the motion of the tissue, high frame rate plane wave imaging created by a specially designed beamformer is used [17,22,26]. The SSI system measures the local shear wave speeds and creates a two-dimensional map representing the shear wave speed distribution. This information is used to obtain an estimated tissue elasticity expressed in units of kiloPascals (kPa) [20]. The studies on the diagnositic value of shear wave imaging using SSI for characterization of breast masses have shown promising results [27,28,29,30,31,32,33,34].

Recently, Song et al. [35,36] have developed an ultrasound shear elastography technique using multiple simultaneous ARF beams called Comb-push Ultrasound Shear Elastography (CUSE). CUSE allows one to obtain a full field of view (FOV) shear wave speed at a high frame rate with a single push-detect data acquisition. Supersonic Shear Imaging requires 3 repeated acquisitions to produce a similar FOV [20]. Therefore, the relatively short acquisition time of CUSE may be beneficial for certain in vivo applications where physiological motions such as cardiac and breathing motions are non-negligible. In CUSE, the ARF beams are spaced laterally for shear wave production. The entire FOV is filled with shear waves travelling in both lateral directions. Using a directional filter, shear waves traveling in opposing directions are separated and used to estimate the elasticity map of the full FOV under the transducer in one single comb-push acquisition [37]. The high frame rate in CUSE allows for a fast acquisition time, which helps avoiding artifacts resulting from physiological activities such as cardiac pulsation and breathing motion. The first *in vivo* shear wave elastography using CUSE for differentiation of thyroid nodules was recently presented [38].

In this study, the clinical utility of CUSE to differentiate between benign and malignant breast masses is investigated. The descriptions of our cohort, as well as the statistical results of the CUSE performance, are presented.

## Materials and Methods

### Patients

Under an approved protocol by the Mayo Clinic Institutional Review Board (IRB), female volunteers with suspicious breast masses on their clinical evaluation were selected for this study. A written signed informed consent with permission for publication, approved by Mayo Clinic IRB, was obtained from enrolled patients. We excluded patients with a history of breast implants and mastectomies. From January 2013 to July 2014, a total of 64 patients were enrolled for the study. The first eight patients were utilized for optimization of the CUSE technique. Two patients were excluded due to hardware issues that occurred during the study. In total, 54 patients were examined with the CUSE imaging technique. All of our patients had received a clinical ultrasound and mammography prior to participating in the study. CUSE was performed prior to biopsy in all cases. When multiple masses were present, only the single mass to be biopsied was interrogated.

### Conventional Ultrasound and CUSE imaging examinations

**Fig 1:**
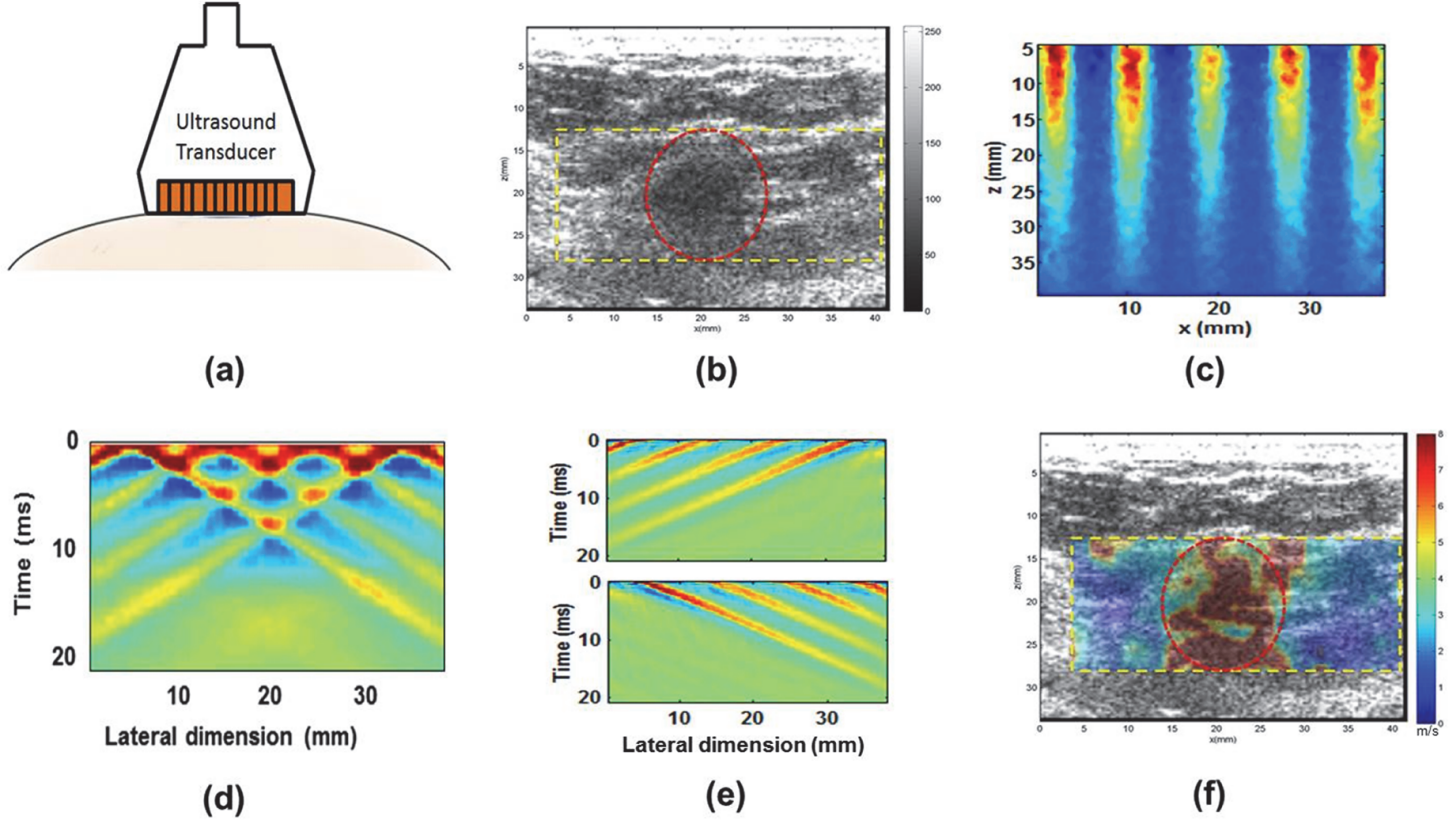
US/CUSE imaging diagram. (a) Probe and patient orientation US/CUSE imaging of the breast mass. (b) Marked US image of the breast mass (red circle). (c) An example of comb push ARF excitation in a phantom. (d) Generated shear waves due in response to the comb-push excitation in the phantom. (e) Directional filters are used to separate LR (Top) and RL (bottom) waves in the phantom. (f) The post-processed shear wave speed is overlaid onto the US image.

CUSE imaging was performed using the Verasonics V-1 system, a fully programmable ultrasound platform (Verasonics Inc., Redmond, WA), equipped with a linear array transducer L7-4 (Philips Healthcare, Andover, MA). The patients were scanned in a supine or lateral oblique position. Conventional B-mode US was first performed to identify the area of the mass. Then, the probe was fixed in place by a lockable articulated arm. This breast mass area was marked on the image by freehand drawing to identify the region of interest (ROI). A highly experienced sonographer performed the examination, and care was taken to minimize pressure on the skin to avoid the possible effect of pre-compression [39]. In order to reduce possible breathing motion artifacts, the patient was asked to suspend respiration for a few seconds for each CUSE measurement. Thereafter, the CUSE was conducted. The US/CUSE procedure is outlined in Fig. 1.

In the CUSE technique, shear waves are produced by multiple laterally-spaced ARF beams (Fig. 1C). The center frequencies of the ARF beams are set to 4.09 MHz with pulse duration of 600 μs. Upon excitation of the tissue, the Verasonics system immediately switches to plane wave imaging mode at a 5 MHz center frequency to track the resulting shear wave propagation. The shear wave particle velocity is tracked by the compounding plane wave imaging method and calculated by the 1-D autocorrelation method using the in-phase/quadrature (IQ) data [36,40,41]. The ARF beams can be either unfocused (UCUSE) or focused (FCUSE) [35] depending on the depth of the mass. For depths greater than 1cm, we solely employ FCUSE with an F-number equal to one for each ARF beam. In the UCUSE technique, the US probe elements are divided into subgroups simultaneously transmitting unfocused push beams. Similarly, in the FCUSE technique, the probe elements are divided equally into subgroups simultaneously transmitting focused ARF beams. An example of the UCUSE ARF push beams are shown in Fig. 1C. The push beams generate shear waves in the tissue, with some waves interfering with each other constructively and destructively, as shown in shown in Fig. 1D. In order to construct a robust shear wave speed map, a directional filter is used to extract the left-to-right (LR) and the right-to-left (RL) propagating shear waves from the interfering waves at each pixel. The directionally filtered waves are shown in Fig. 1E. Thereafter, a time-of-flight algorithm based on cross-correlating shear wave motion profiles along the lateral direction was used to calculate shear wave propagation speed. The shear wave speed of an imaging pixel was calculated from two neighboring pixel points at the same depth [40]. In Fig. 1F, a final shear wave speed map (averaging LR and RL speed maps) is color coded and overlaid onto the B-mode US image. Quantitative measurements of tissue elasticity are obtained from the ROI. Additional technical information of CUSE shear wave speed calculation and shear wave speed map reconstruction are detailed elsewhere [35].

### Image analysis and classification

A graphical user interface was developed using Matlab (MathWorks Inc., MA, US) to process acquired CUSE data and to reconstruct the shear wave speed map. The processing includes using the normalized cross-correlation coefficient of the shear wave speed map as a quality control factor [42] to reject pixels with unreliable speed measurements, as well as applying a *n×n* (*n* = 3 or 5) mean filter to smooth the shear wave speed map. Each CUSE image was acquired in approximately 25 ms; no denoising or frame averaging was performed. The shear wave speed map is displayed by a color map indicating shear wave speeds ranging from 0 to 8m/s. Tissue stiffness estimates are obtained within the ROI from the overlaid shear wave speed map. The elasticity value of a breast mass is calculated as the Young’s modulus of the mean shear wave speed within the ROI. Assuming a linear, isotropic, incompressible and elastic soft-tissue the Young’s modulus is obtained from the expression

$$E=3\rho c_{s}^{2}$$ (1)

where *ρ* = 1000 kg/m^3^ represents the tissue density and *c_s_* is the shear wave speed. In each case, the pathology of the masses was determined by clinical biopsy.

### Statistical analysis

In order to assess the performance of CUSE, the receiver operating characteristic (ROC) analysis was performed. An optimal cut-off value, which maximizes the sensitivity and specificity of the ROC curve, was established. These statistical measures of the CUSE performance are specific for this study population. The significant differences in categorical variables between benign and malignant groups were assessed using the Mann-Whitney U test. Two-tailed *P* values of less than 0.05 were considered to indicate statistical significance. Statistical analyses were performed with MATLAB software.

## Results

We present the CUSE results for 54 pre-biopsy female breast patients. The pathological diagnoses were 23 benign and 31 malignant masses. The tumor sizes of the malignant masses (0.4 to 6.6 cm) were greater than those of benign masses (0.5 to 3.5 cm) in the greatest dimension. The benign and malignant histology distribution and BI-RADS classification of the breast masses are summarized in Table 1.

**Table 1:** Histology of 54 breast patients.

Histopathology	BIRADS 2	BIRADS 4	BIRADS 5
Malignant (n = 29)	0	12	19
Benign (n = 23)	1	21	1

**Fig 2:**
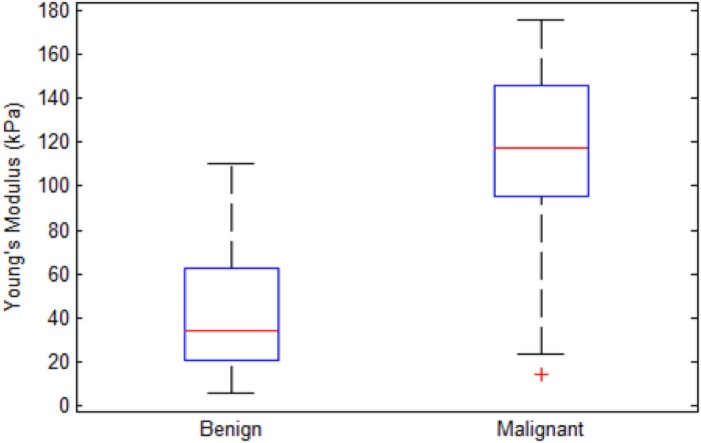
Box-and-whisker plots of SWE values for benign and malignant breast tissue. Young’s modulus values are reported on the y-axis and breast tissues were reported on the x-axis. The central box represents values from the lower to upper quartile (25^th^–75^th^ percentile). The line through each box represents the median. Error bars show minimum and maximum non-extreme values. ‘+’, are extreme values.

**Fig 3:**
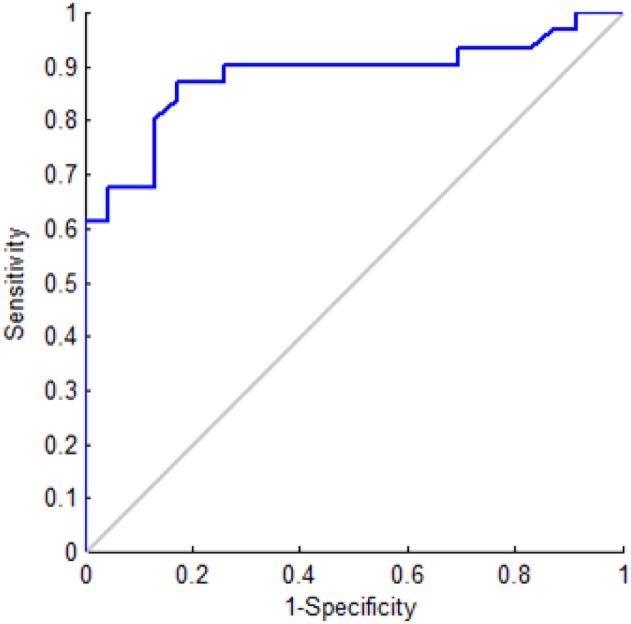
ROC curve of the CUSE imaging technique. The diagonal line is the line of no-discrimination.

Benign and malignant breast masses showed a mean shear wave speed of 3.65 ± 1.36 m/s and 6 ± 1.58 m/s (*P<0*.*0001*), respectively. The corresponding Young’s modulus statistics for the benign and malignant masses are shown in Fig. 2. The median, interquartile range (25th-75th percentile) and number of outliers for the boxplot results are given in Table 2. The ROC graph, shown in Fig. 3, has a 0.882 area under the curve (AUC). According to the ROC analysis, the optimal Young’s modulus cut-off value >83 kPa yields 87.10% sensitivity and 82.61% specificity. The results of applying the cut-off value to our dataset, in order to downgrade suspicious masses (< 83 kPa), are given in Table 3.

**Table 2:** Median values, interquartile range (IQR) and Number of outliers.

	Benign	Malignant
Median, kPa	34.47	117.56
IQR, kPa	41.56	50.81
Number of Outliers	0	1

**Table 3:** Downgrading of suspicious breast masses.

	Downgrade < 83 (kPa)
True positive	27
False positive	5
True negative	18
False negative	4

### Review of selected cases

The results of six patients are individually reviewed to demonstrate the feasibility of the CUSE imaging technique for detection and differentiation of benign and malignant breast masses.

**Fig 4:**
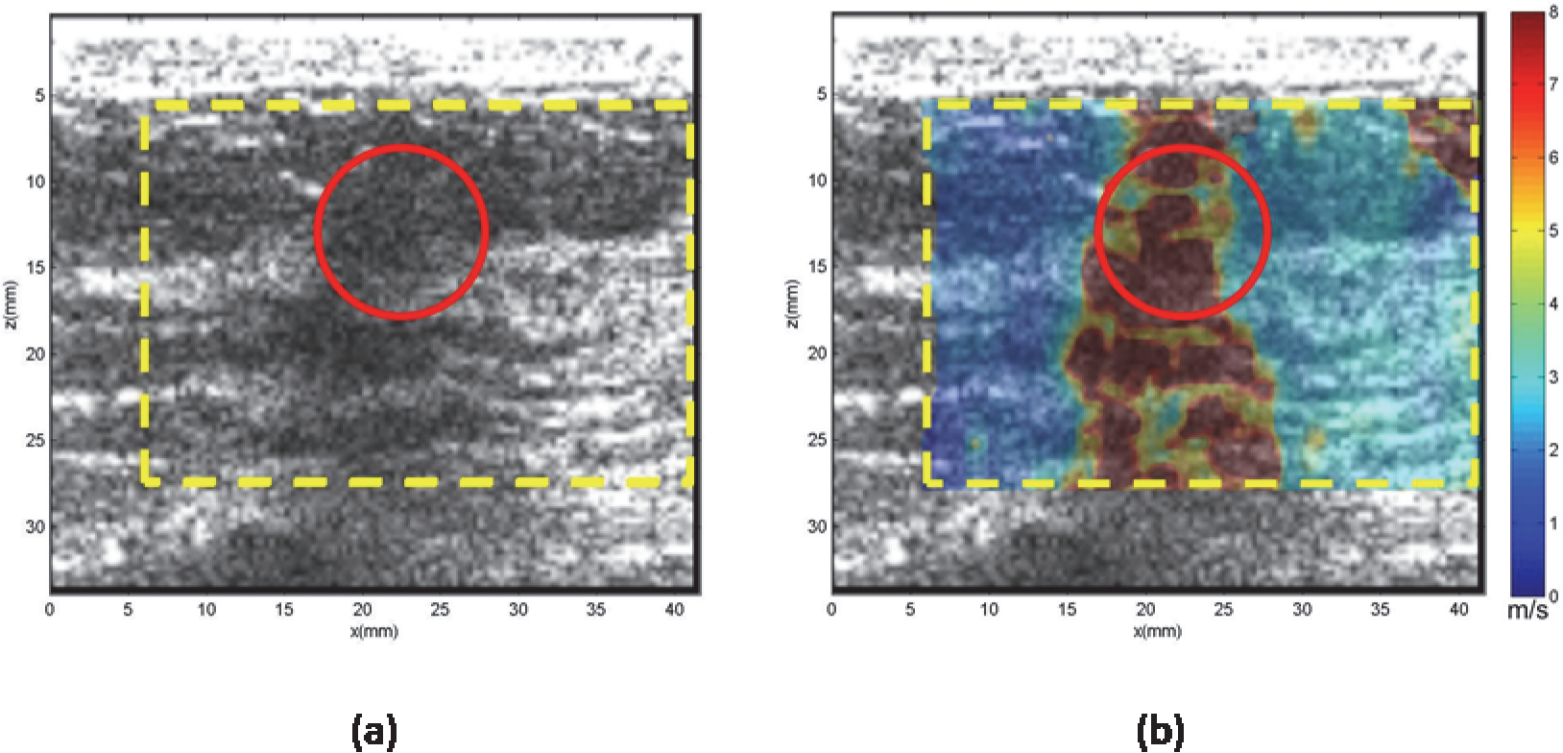
US and CUSE shear wave speed map of breast tissue (dashed yellow contour) including the breast mass ROI (red contour). (a) B-mode US image. (b) CUSE shear wave speed map. The vertical extensions of the high shear wave speeds along the mass location demonstrate the effects of calcifications.

**Case 1.** In Fig. 4, the patient was in her 40s with a palpable mass in her left breast 1 cm in depth. Clinical ultrasound demonstrated a mass measuring approximately 6 mm in the greatest dimension with heterogeneous echogenicity. The CUSE mean and standard deviation of the shear wave speed within the ROI was measured as 7.60 ± 0.84 m/s, which yields a Young’s modulus of 101.6 kPa. Subsequent biopsy results indicated the mass to be benign organizing fat necrosis and abundant hemosiderin-laden macrophages, as well as dystrophic calcifications in stroma.

**Fig 5:**
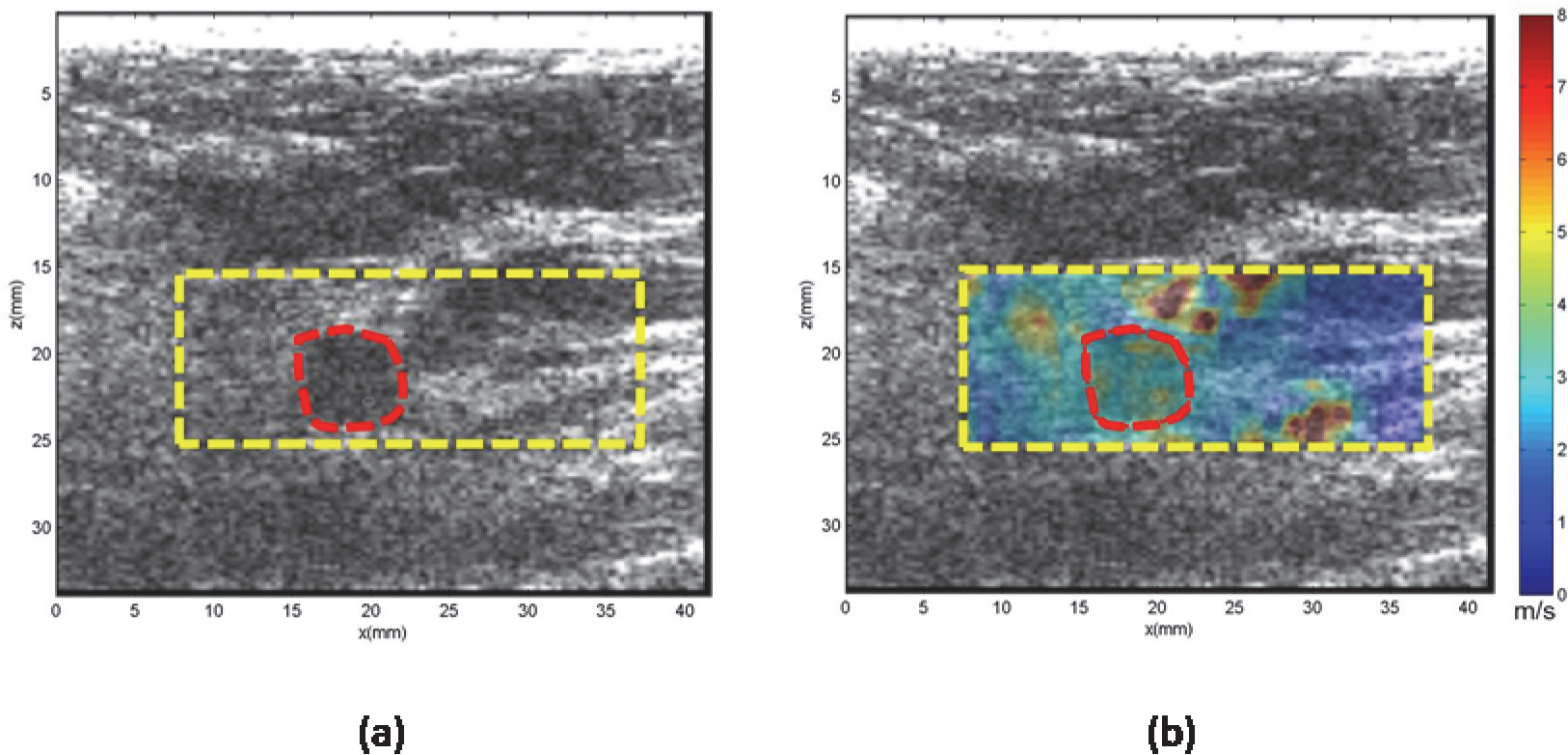
US and CUSE shear wave speed map of breast tissue (dashed yellow contour) including the breast mass ROI (red dashed contour). (a) B-mode US image. (b) CUSE shear wave speed map.

**Case 2.** In Fig. 5, the patient was in her 40s with a mass in her right breast located 2 cm from the skin. Targeted ultrasound revealed a 0.8 cm breast mass in the greatest dimension. The mean shear wave speed of the ROI was measured as 3.21 ± 1.9 m/s, yielding a Young’s modulus of 30.9 kPa. Biopsy results indicate the mass to be benign fibrocystic changes.

**Fig 6:**
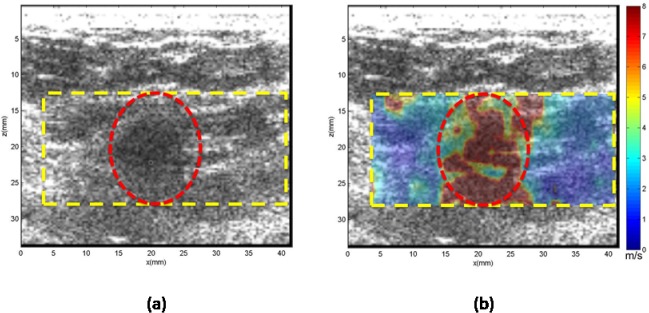
US and CUSE shear wave speed map of breast tissue (dashed yellow contour) including the breast mass ROI (red dashed contour). (a) B-mode US image. (b) CUSE shear wave speed map.

**Case 3.** In Fig. 6, the patient was in her 70s with a mass in her left breast 2 cm in depth. Diagnostic mammography identified scattered fibro-glandular densities in the left breast and reveals a 1.2 cm mass in the greatest dimension. Ultrasound confirmed a 1.2 cm hypoechoic mass. The mean shear wave speed of the ROI was measured as 6.88 ± 1.54 m/s, yielding a Young’s modulus 142 kPa. Subsequent biopsy results revealed the mass to be malignant Nottingham grade II/III invasive ductal carcinoma.

**Fig 7:**
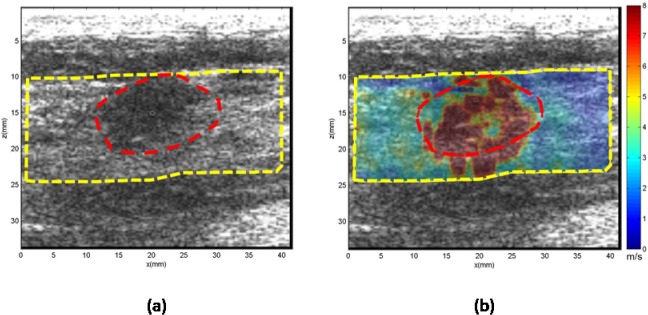
US and CUSE shear wave speed map of breast tissue (dashed yellow contour) including the breast mass ROI (red dashed contour). (a) B-mode US image. (b) CUSE shear wave speed map.

**Case 4.** In Fig. 7, the patient was in her 70s with a palpable lump in her right breast 1.5 cm in depth. Targeted ultrasound of the right breast showed a hyperechoic mass measuring 2.3 cm in greatest dimension. The mean shear wave speed of the ROI was measured as 7.07 ± 1.18 m/s, yielding a Young’s modulus of 149.9 kPa. Subsequent biopsy results revealed the mass to be malignant grade II invasive ductal carcinoma with adjacent ductal carcinoma *in situ*. Calcifications were present in both ductal carcinoma *in situ* and invasive carcinoma.

**Fig 8:**
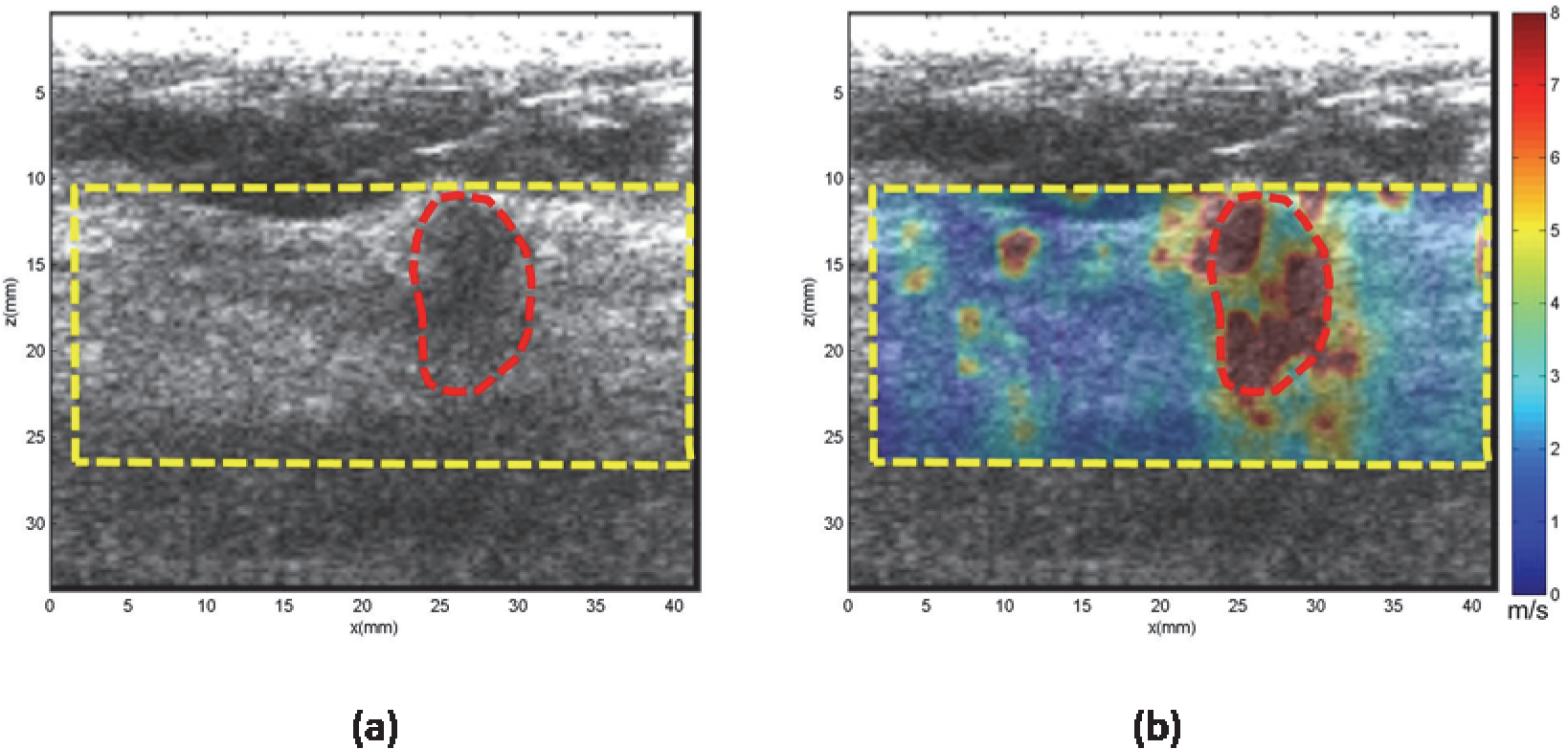
US and CUSE shear wave speed map of breast tissue (dashed yellow contour) including the breast mass ROI (red dashed contour). (a) B-mode US image. (b) CUSE shear wave speed map.

**Case 5.** In Fig. 8, the patient was in her 70s with an irregular mass in the right breast 1.5 cm in depth. Targeted ultrasound demonstrates a 0.80 cm hypoechoic mass. The mean shear wave speed within the ROI was measured as 6.42 ± 1.70 m/s, yielding a Young’s modulus of 123.6 kPa. Biopsy results revealed the mass to be malignant grade I invasive ductal carcinoma.

**Fig 9:**
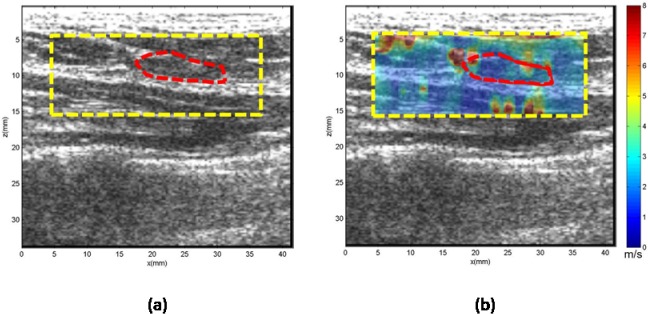
US and CUSE shear wave speed map of breast tissue (dashed yellow contour) including the breast mass ROI (red dashed contour). (a) B-mode US image. (b) CUSE shear wave speed map.

**Case 6.** In Fig. 9, the patient was in her 70s with an irregular mass in the right breast 1 cm in depth. Targeted ultrasound demonstrates a 1.4 cm mass in the greatest dimension. The mean shear wave speed within the ROI was measured as 2.63 ± 1.59 m/s, yielding a Young’s modulus of 20.75 kPa. Biopsy results revealed the mass to be a benign fibroadenoma.

## Discussion

This paper presents the results of a new shear wave elastography method called CUSE. There are some differences between CUSE and the Supersonic Shear Imaging (SSI), a well-known shear wave elastography, which is currently in clinical use. The main difference between the two methods is the way the push beams are applied to the tissue. The SSI method uses multiple sequential push beams focused at different depths at almost identical lateral positions to constructively produce shear wave fronts over an extended range in depth. The CUSE method, on the other hand, uses multiple simultaneous push beams focused at the same depth but laterally spaced apart. These beams generate multiple interfering shear waves, which are decomposed into unidirectional shear waves by directional filtering. Subsequently, shear wave speed calculation is performed [35,36]. The focus of this paper was to evaluate the diagnostic value of CUSE in breast cancer detection. For this reason, we did not intend to elaborate on comparing CUSE with SSI or other shear wave elastography methods.

In our patient population, the majority of malignant masses showed higher stiffness values than benign masses. In Fig. 2, there is clear separation of the median Young’s modulus between benign and malignant masses. Our ROC curve has an optimal cut-off value (> 83.00 kPa) concordant with those in previous studies [29,43] for suspicious breast mass. Berg et al. [43] studied 939 breast masses and defined masses with elasticity value > 80 kPa as suspicious, achieving a sensitivity of 97.2%, specificity of 77.4%, PPV of 65.7% and 0.959 AUC. Chang et al. [29] studied shear wave elastography for 182 breast masses (89 malignant, 93 benign) with an optimal cut-off value of 80.17 kPa, achieving 88.8% sensitivity, 84.9% specificity, and 0.932 AUC. Since these previous studies include masses classified as BI-RADS 3, and ours is mainly composed of BI-RADS 4 and 5, direct comparisons of the ROC curves are not completely accurate.

Overlap does occur in our results between the Young’s modulus values of the benign and malignant datasets, as shown in Fig. 2. In Table 3, the overlap results in five false positives and four false negatives. The false negatives were composed of masses with a ROI cross-section of less than 0.49 cm^2^ (*n* = 2), and masses with a noticeable high stiffness region but smaller than the ROI (*n* = 2) cross-section. The false positives were composed of masses with calcifications (*n* = 2), complex sclerosing (*n* = 1), atypical ductal hyperplasia (*n* = 1) and granulomatous inflammation (*n* = 1). Masses with calcifications are known to induce false positives [44]. Fig. 4 demonstrates the effect of calcifications on the shear wave speed map with vertical regions with high shear wave speed values along the position of the mass. Also, masses that have a structure with a firm consistency, such as complex sclerosing, can induce false positive results. Further investigation, as well as additional data, is required for the remaining false positive and negative cases.

Future studies can be conducted to advance this study in several different directions. First, one can utilize a larger cohort to study the performance of this method on subtypes of benign and malignant masses. Second, one study can be dedicated to assess inter-observer variability. However, shear wave elastography techniques have been shown to be highly reproducible [31]. Third, correction for the effect of pre-compression on elasticity estimation of breast masses requires further studies. Pre-compression of tissue has been shown to increase the measured shear wave speed [39]. The pre-compression effect on elasticity has been previously investigated [12,45]. Changes in shear wave speed due to static stress are related to the nonlinear elasticity of the material and can be described under the acoustoelasticity theory [46]. Latorre-Ossa et al. [47] quantified the pre-compression effect on shear wave speed from the nonlinear elastic behavior of soft materials. This theory describes the relation between shear wave speed, the stress-free shear elasticity, a parameter of nonlinearity, and the applied stress. Using this theory, investigators have calculated the stress-free shear modulus and the nonlinear parameter A of tissue [47]. Recently, Barr et al. [39] have presented a semi quantitative method to account for pre-compression on shear wave elastography. In the present study, we applied minimal pressure on the probe just to make contact with the skin; hence, we did not include pre-compression effect in our analysis.

## Conclusions

In conclusion, the CUSE imaging technique showed a significant difference with respect to the mean shear wave speed between benign and malignant masses. Our results were comparable to previous SWE studies of suspicious masses, with a Young’s modulus > 83 kPa as our optimal cut-off value. Therefore, the CUSE imaging technique may be useful as a noninvasive method as an adjunct to breast ultrasound for differentiating benign and malignant breast masses, and may help in reducing the number of unnecessary biopsies.
